# Is that SMART? Leveraging Collaboration and Peer Feedback to Enable Practice in Appraising and Presenting Journal Articles

**DOI:** 10.1007/s40670-024-02188-5

**Published:** 2024-10-15

**Authors:** Mary Kate Worden

**Affiliations:** Office of Medical Education, Center for Medical Education Research and Scholarly Innovation, University of Virginia School of Medicine, Charlottesville, VA USA

**Keywords:** Journal article, Journal club, Narrative feedback, SMART

## Abstract

In an innovative journal club exercise, groups of students collaboratively appraise and present a journal article and then provide SMART narrative feedback to a peer group on their presentation. Feedback is exchanged between groups that presented the same journal article, increasing the likelihood that feedback will be meaningful and high-quality.

Staying up-to-date with the scientific literature is critical for healthcare providers because new research findings can stimulate re-evaluation of current diagnostic and therapeutic practice [1]. Medical journal clubs provide useful venues for knowledge discovery and dissemination by requiring participants to discuss the substance of a selected journal article, as well as their own appraisal of it, with colleagues [2]. Journal club exercises also provide valuable opportunities for learners to practice crafting and delivering effective audiovisual presentations.

For students to improve the analytical and communication skills they practice in journal club, they must receive meaningful feedback on the strengths and weaknesses of their efforts. Providing this feedback can require significant faculty time and effort and may not be feasible in situations where there are large numbers of learners relative to instructors.

Here we describe an innovative journal club exercise that leverages the possibility that peer feedback can be effective in improving students’ presentation skills [3].

Second-year medical students (*n* = 160) were assigned randomly into 40 groups of four students. Each group was assigned to read and present one of three journal articles from the epidemiology literature. Students received 10 min in-person instruction on the principles of providing narrative feedback that was SMART [4], i.e., specific (descriptive of what the presenter did), measurable (quantifiable), attainable (focused on behavior, not personality), relevant (to giving audiovisual presentations), and time-bound (useful for in the next journal club exercise). Students had 3 days to prepare an audiovisual presentation of the article in which each group member presented at least one of five slides covering Background, Methods, Results, Discussions/Limitations, and Summary. A Zoom recording (of 15 min or less) was made by each group and uploaded to the Learning Management System so that others could access the recordings for review. Each group had 50 min of class time to watch the recording produced by another group and provide narrative SMART feedback to them on the quality of their presentation. Feedback was collected using a Qualtrics survey form and returned to the other group by email. To increase the likelihood that the feedback would be meaningful, all groups were assigned to review the presentation of another group that had read and presented the same journal article. The exercise culminated in a large group discussion in which a panel of experts (epidemiologists and pulmonary and critical care specialists) presented their own critiques, thereby enabling students to recognize their strengths and weaknesses in their appraisal of the article. The schematic in Fig. 1 demonstrates the exchange of presentations and feedback among student groups.

**Fig. 1:**
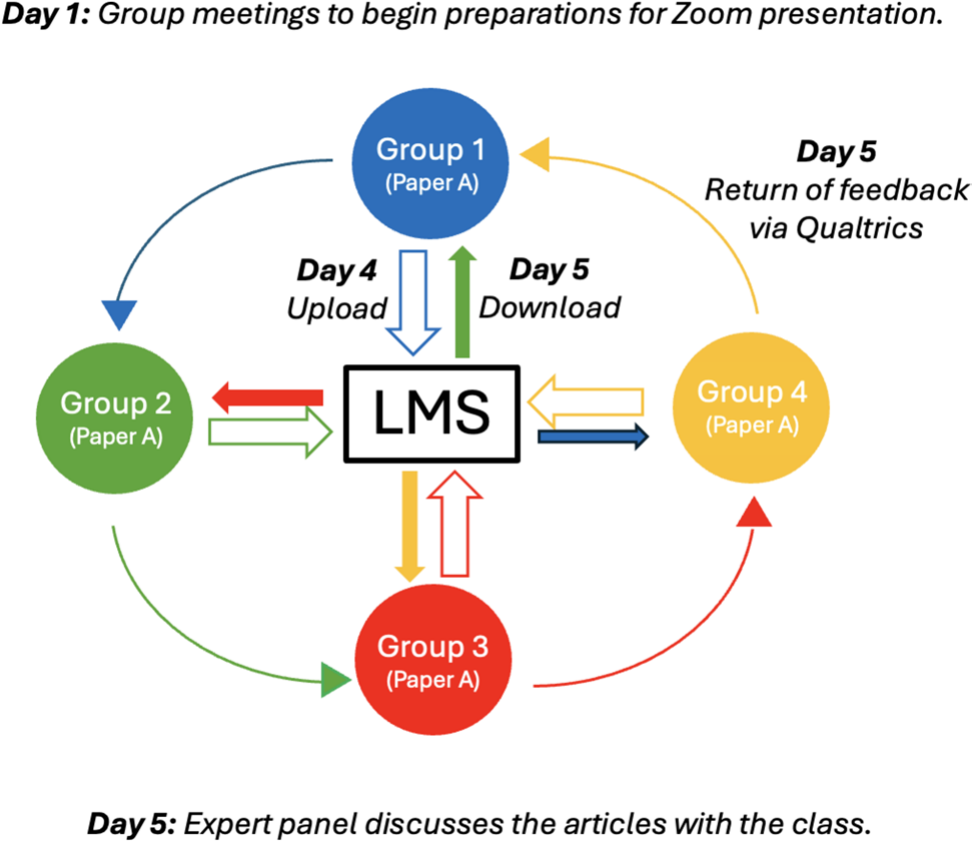
After meeting on day 1 to plan, student groups uploaded their presentations (open arrows) to the Learning Management System (LMS) by day 4. On day 5, each group downloaded and reviewed a presentation by another group about the same paper (closed arrows) and provided SMART feedback to their peers via Qualtrics (curved arrows). Here, group 1 provided feedback on group 2’s presentation; group 2 provided feedback on group 3’s presentation, etc. Day 5 concluded with a class discussion of all papers

The percentage of students who reported on a post-class survey that the peer feedback they received was Specific, Measurable, Attainable, Relevant, and Time-bound was 89%, 68%, 83%, 80%, and 56%, respectively. An overwhelming number of comments regarding the feedback (*n* = 150) on the post-class survey were positive. Selected narrative comments from students are shown in Table 1.

**Table 1:** Student commentary on post-class survey

• Getting feedback from someone who had also read the same article in depth and done their own presentation on it was surprisingly helpful. It gave the feedback greater credibility and I think allowed the other group to be more specific • I found the feedback provided to my group incredibly helpful. It included specific elements of the presentation that should be improved and provided ideas for improvement • Providing feedback on what worked helps almost as much as the SMART advice on what could be improved. It shows the foundation I can build on • The most helpful part was gaining specific and time-specific feedback that we can focus on to improve our presentation skills in the future			

Several elements of this initiative were likely critical to its success. First, posting the recorded presentations to the course website uncoupled the timing of the feedback from the timing of the presentation, thereby eliminating any need to co-schedule presenters and reviewers in the same place and time. Second, providing students with a brief orientation to SMART feedback (and including the definition of the SMART acronym on the feedback form) encouraged reviewers to provide feedback that recipients recognized as high-quality. Third assigning students to review a presentation of the same journal article enabled “spaced repetition” of the content of the article, and likely increased the specificity of the comments on the reviews. Fourth, scheduling in-class time for groups to collaboratively craft the SMART feedback made all group members accountable to each other for ensuring that feedback was high quality.

In summary, this journal club exercise successfully enabled a class with a low ratio of instructors/students to practice key analytical and communication skills because it was structured around collaboration, recordings of presentations, certainty that reviewers and presenters had read the same article, a brief orientation to the qualities of SMART feedback, and accountability within groups for providing high-quality feedback to peers.

## Data Availability

The participants of this study did not give written consent for their data to be shared publicly, so due to the sensitive nature of the research supporting data is not available.

## Declarations

This is an observational study. The Institutional Review Board reviewed and exempted this project from further review (Protocol #6544).

## References

[1] du Prel J-B, Röhrig B, Blettner M. Critical appraisal of scientific articles: part 1 of a series on evaluation of scientific publications. Dtsch Arzteblatt Int. 2009;106(7):100–5. 10.3238/arztebl.2009.0100. 10.3238/arztebl.2009.0100 PMC2696241 19562021

[2] Meleger AL, Co JPT, Zafonte RD. Rethinking medical journal club. Am J Med. 2020;133(5):534–5. 10.1016/j.amjmed.2019.10.033. 31743658 10.1016/j.amjmed.2019.10.033

[3] Murillo-Zamorano LR, Montanero M. Oral presentations in higher education: a comparison of the impact of peer and teacher feedback. Assess Eval High Educ. 2018;43(1):138–50. 10.1080/02602938.2017.1303032.

[4] Finkelstein SR. “SMART feedback – how to design and provide effective feedback,” The Learning Scientists. Accessed: Aug. 25, 2024. [Online]. Available: https://www.learningscientists.org/blog/2017/4/19-1

